# Increasing opportunities for community input in harm reduction program development using iterative engagement

**DOI:** 10.1186/s12954-025-01323-8

**Published:** 2025-10-22

**Authors:** Nicole M. Wagner, Jordan A. Carlson, Meagan Bean, Amy Wineland, Joshua Blum, Scott A. Cardona, Sheila Covarrubias, Allison Kempe, Abby C. King, Amy G. Huebschmann

**Affiliations:** 1 Adult and Child Center for Outcomes Research and Delivery Science (ACCORDS), University of Colorado Anschutz Medical Campus, 1890 North Revere Court, Aurora, CO 80045 USA; 2 Division of General Internal Medicine, School of Medicine, University of Colorado Anschutz Medical Campus, 12631 E 17th Ave., Aurora, CO 80045 USA; 3 Center for Children’s Healthy Lifestyles & Nutrition, Children’s Mercy Hospital, 2401 Gillham Rd., Kansas City, MO 64108 USA; 4 Summit County Public Health Department, 360 Peak One Dr, Frisco, CO 80443 USA; 5 Center for Health Systems Research, Denver Health Hospital and Authority, 777 Bannock St., M.C 6551, Denver, CO 80204 USA; 6 Department of Pediatrics, School of Medicine, University of Colorado Anschutz Medical Campus, 13123 E. 16th Ave. B065, Aurora, CO 80045 USA; 7 Department of Epidemiology and Population Health, Stanford University School of Medicine, 1701 Page Mill Road, Palo Alto, CA 94304-1210 USA; 8 Ludeman Family Center for Women’s Health Research, School of Medicine, University of Colorado Anschutz Medical Campus, 12631 E 17th Ave., Aurora, CO 80045 USA

**Keywords:** Community engagement, Implementation science, Harm reduction, Substance use, Iterative engagement, Environmental evaluations

## Abstract

**Background:**

Incorporating people who use substances into a community-engaged research process can support the implementation and evaluation of evidence-based harm reduction programs. Attending to their voice ensures those who need these programs will use them. Yet, ongoing co-learning with people who use substances, often the ideal for community engaged research, poses a challenge for recruitment, ongoing participation, and obtaining diverse perspectives. We need novel strategies to support flexibility among populations experiencing legal and social instability so that community engaged work includes more diverse perspectives. In this paper, we describe a novel community engagement approach called Effective Adaptable and Sustainable in Your Community: Operationalizing Program Sustainability (EASY OPS). EASY OPS uses iterative engagement with people with lived/living substance use experience to design and implement harm reduction vending machine and kiosk programs, aiming to increase program use in those who would benefit most.

**Main body:**

The EASY OPS approach addresses two key challenges to access and use of evidence-based harm reduction programs in underrepresented populations: (1) the need for attention to elements of the environment, and (2) ways to navigate challenges to ongoing research collaboration with community members experiencing substance use disorders. EASY OPS uses walking interviews with participants to identify environmental factors contributing to perceived use of services. Iterative engagement with community members–through interviews, surveys, and focus groups–was conducted to inform program development from the community’s perspective as feasibility challenges emerged.

**Conclusions:**

This paper describes the novel EASY OPS strategy that facilitates iterative community engagement for harm reduction research and program development to better tailor implementation to the needs of diverse populations with lived/living experience. The potential impact is to reduce disparities by enhancing representative reach and access to substance use service and harm reduction programs.

## Background

Evidence-based programs (EBPs) for harm reduction have been used across the world for decades, including overdose education and naloxone distribution (OEND) and syringe service programs [1]. These harm reduction programs have demonstrated effectiveness at reducing negative health outcomes including overdose prevention, HIV/HCV transmission, and bacterial infections [2–5]. Additionally, they are cost-effective, have increased use of treatment and social services, and have not increased risk behaviors [6–10]. Despite the evidence demonstrating successful outcomes of these harm reduction EBPs, implementation has been limited and inequitable in the U.S [11]. This is highlighted by the current gaps in translation of research to practice and equitable community access to substance use and harm reduction services [12, 13]. As a result, it is imperative to better engage community members in the design, planning and implementation of harm reduction programs to ensure they are accessible and tailored to the contextual needs of those who will use them.

Community Based Participatory Research (CBPR) is a research approach designed to equitably involve community members in the research process [14]. CBPR for program implementation includes diverse expertise in which researchers, organizations implementing programs, community members, and other relevant partners work together to generate knowledge, learn from each other and facilitate change [15]. Common themes of CBPR include fostering collective-decision making in identifying health priorities, recognition and appreciation for priorities of all involved partners, building capacity for community members and researchers to engage in research collaboratively, and recognizing research is not the endpoint but requires ongoing commitment for dissemination [16]. The level of community engagement in CBPR can vary, but ideally includes building and sustaining genuine partnerships with community members in order to obtain meaningful input throughout the process [15]. However, achieving this can be difficult due to extended timelines and funding schedules [17]. A study found more than 60% of investigators and research staff identified time commitment as a key barrier for community member participation in CBPR work [18]. 

Ongoing community engagement can be particularly challenging in harm reduction work. Peer-led organizations often lack dedicated, flexible funding to support their involvement in recruiting or sustaining peers [19]. Similarly, evidence suggests CBPR involving people with lived or living substance use experience (hereafter PWLE) faces several barriers, including unreliable phones and transportation, difficulties in obtaining diverse perspectives within the drug user communities—often due to stigma and tendency of peer recruiters to recruit participants similar to themselves—and high dropout rates driven by social and legal instability [20, 21]. Rural settings add barriers such as challenges identifying local trusted partners and over-reliance on key individuals juggling different community roles [22]. A scoping review of CBPR work with PWLE recommended a more flexible approach to CBPR by allowing practices that are more responsive to the sometimes chaotic living conditions of low-income people who use drugs to allow for more inclusion and incorporation of diverse perspectives [21]. 

In addition to the need for efficient strategies that support fluid participation to obtain ongoing and diverse input from PWLE, community perspectives on the features of the local environments suitable for harm reduction services have been critically overlooked in most harm reduction research to date [23]. Access to harm reduction services, including community availability in high-traffic areas near transportation routes, is a well-established macro-scale environmental factor contributing to use of services [24]. However, micro-scale attributes of the environment observable to a potential user (e.g. visible security guards, lighting, roles of people in the environment–such as customers or staff) [25], may have a similar impact on use of services and are often not considered. For example, young adults who use opioids reported privacy as a key barrier to accessing naloxone and preferred discrete locations for naloxone vending machines [26]. While security cameras were noted as a positive feature that provides a feeling of safety, a security guard presence was reported as a strong deterrent for use of naloxone vending machines. Even if the implementation team cannot change the location of services, understanding the environmental features that can deter use of services allows for necessary adjustments to increase equitable service utilization.

In this article, we describe a novel community-engaged process called “Effective Adaptable and Sustainable in Your Community: Operationalizing Program Sustainability (EASY OPS).” This approach supports sustained engagement by having the research team mediate iterative collaboration between PWLE and the implementation team. Together, harm reduction programs that reflect the needs of both end users and service providers, thereby reducing access barriers to substance use services. We describe the EASY OPS process in the development of a VEnding machine Naloxone Distribution program in Your community (VENDY) program. The EASY OPS collaboration included the research team, the implementation team responsible for program delivery at each site, and community members reporting illegal substance use in the last year, indicating current or recent lived experience (PWLE). EASY OPS incorporated PWLE feedback through environmental assessments to identify contextual program needs. To be efficient in our engagement approaches, we supported the involvement of unique PWLE to provide insightful and timely feedback over the course of the project. In terms of representativeness of these settings for others’ work, we conducted VENDY in three diverse settings: an urban health system, an urban harm reduction agency, and a public health department in a rural mountain community. The overall objective of this article is to examine how EASY OPS and other novel community engagement strategies may address two key challenges to access and use of substance services in underrepresented populations: (1) the need for attention to elements of the environment impacting use of services, and (2) ways to navigate challenges to sustained and diverse engagement, as PWLE are often unwilling or unable to participate consistently across the research life cycle.

## Harm reduction intervention: VENDY

Harm reduction vending machines (HRVMs) have been used for over 40 years in communities across the world, but have only recently been implemented in the United States [27]. The number of HRVM programs has expanded since COVID-19 due to no contact requirements and rising opioid overdose events [28]. HRVM programs can vary, but always include harm reduction supplies, often naloxone, in a vending machine or newspaper kiosk. HRVM programs typically give supplies to community members at no cost. Some HRVMs require user registration to access supplies depending on the types of supplies in the machines (e.g., syringes), state laws, and local organization requirements. Machines may be placed on sight at an organization, but are often placed in partnership with another organization whereby one is overseeing the program and the other agrees for the machine to be placed on site [27]. There is overwhelming evidence for the effectiveness of naloxone to prevent opioid overdose deaths [3], and early evidence to suggest that HRVM programs, specifically, are effective at reducing opioid overdose deaths [29]. Each of the three participating sites involved in the present study implemented the VENDY program, which included a vending machine or kiosk for naloxone distribution, overdose education materials, and iterative engagement with PWLE as described below.

## Sites, participants and recruitment

The research team, including an implementation and substance use scientist (NW) and program manager (MB), conducted this work with three organization partners in Colorado: an integrated safety-net institution in an urban location in Colorado, a harm reduction agency in an urban Colorado location serving a predominantly Hispanic/Latinx population, and a public health department in a rural mountain town with high tourist presence in the winter months. Each participating site had an implementation team including a department or organization leader and staff responsible for program implementation. Additional organization and community leaders were involved in decision making as appropriate for the organization and at different stages in the iterative process.

To participate, community members had to reside in the respective community, be at least 18 years of age or older, and report use of substances that may contain opioids in the last year. The research team recruited community members in collaboration with the implementation team, using a convenience sample from organizations that provide substance use services in each of the 3 respective communities. Recruitment fliers and emails contained opt-in procedures so that interested participants could contact the research team. Implementation teams distributed materials to community organizations and substance use program participants. Additionally, the research team used snowball sampling, encouraging participants to refer eligible peers. We provide additional details for recruitment in the results for each participating site below. To protect anonymity, we obtained a waiver of signed informed consent. Interviewers obtained verbal consent, and survey participants agreed to consent online, with research team contact information provided for questions. Participants could voluntarily provide contact information in online surveys to receive an incentive.

## Framework for community engagement

This novel EASY OPS community engagement process incorporates principles from two methodologies, user centered design (UCD) and the *Our Voice* citizen science method. UCD is a methodology often used in the development of technologies, with growing use in other disciplines [30, 31]. UCD includes feedback at multiple time points from multilevel partners during program development [31, 32]. Specific to community member participation, end-user input is obtained when the initial ideas are developed, as mock-ups are designed, and again in the pilot testing phase. The same end-users can, but are often not, included in different stages of product development. Additionally, the ways in which end-users participate can vary as needed to address the specific phase and needs of program development. This can include interviews, document reviews, focus groups, usability testing, and observations [33]. Iterative, diverse input supports the development of products that will be used and sustained.

*Our voice* is an evidence-based scientific engagement method. Developed by Dr. King and colleagues at Stanford University, *Our Voice* actively engages residents as citizen scientists in health promotion research through systematic data collection, interpretation, and community solution building to advance local opportunities for health-enhancing environmental and policy change [34]. There are 4 major steps in the *Our Voice* method: (1) Discover, in which community members use the multi-lingual Discover Tool mobile application (app) to take photos and record insights as they walk their community and identify aspects of the environment impacting health living; (2) Discuss, in which community members, in a facilitated process, share findings with other citizen scientists and prioritize target areas for change; (3) Activate, in which citizen scientists present their findings to relevant decision makers in developing feasible solutions; and (4) Change, the final step in which the citizen scientists, working with local decision makers, track results and participate in changing their community for the better. For this project, we used components of the Discover, Discuss, and Activate steps in our community engagement process, supporting assessment of environmental features impacting perceived use of naloxone vending machines. As a program development project, the Change step was not yet applicable.

## Novel community engagement strategy: EASY OPS

**Fig. 1:**
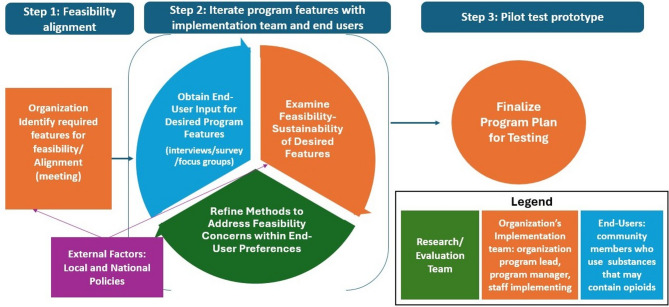
Effective, adaptable and sustainable for your community: operationalizing program sustainability (EASY-OPS)

Researchers combined *Our Voice* and UCD to develop our community engagement strategy, called Effective Adaptable and Sustainable in Your Community: Operationalizing Program Sustainability (EASY OPS) (Fig. 1). The iterative process included: (1) feasibility alignment with the implementation team (defined as the researchers identifying program restrictions and initial thoughts on potentially feasible placement locations); (2) iterative development of program features and locations with the community members and implementation team; and (3) development of a final plan (prototype) for pilot testing in each location. The iterative program development incorporated additional methods used to engage community members, which varied somewhat based on the needs of each respective site (e.g., interviews, focus groups, surveys). Additional details on the methods used and the contextual factors contributing to the choice of methods can be found in each case example.

Feasibility alignment began with the implementation team identifying any program restrictions with respect to the macro-environment of machine placement (e.g., entire community, specific city, health campus) and features (e.g. temperature controls) and exploring initial thoughts on feasible placement. Maps used in initial interviews with community members were based on the feasible areas for placement. The research and implementation team then recruited community members for interviews, as described in the recruitment section above. Each interview (n = 16) lasted approximately 2 h and participants received $75 for their time ($25 for online surveys).

Resident data collection was based on the *Our Voice* method, adapted slightly by conducting the Discovery Tool walk with a trained interviewer. For the Discover step of *Our Voice*, participants received a map of feasible placement areas and were asked to identify the top 5 and bottom 5 locations for machine placement and reasons why they made their selections. This step addressed the macro-scale attributes of the environment including the layout of the community such as access to transit stations. The interviewer and participant then walked or drove to the top 5 areas and discussed environmental features that may influence their use of the machines for naloxone at that location. This represents the evaluation of micro-scale attributes including discussing the natural (e.g. weather, foliage), built (e.g. buildings, benches, sidewalks) and social (e.g. types of people in the area) environmental factors contributing to perceived use of the machine. The interviewer asked participants to take photos of the environmental features on the walk/drive using the Discovery Tool app [35]. In the final step of the data collection process, the interviewer asked participants to reflect on their walk and to identify the top locations they recommended for placing the machine (Discuss step of *Our Voice*) and capture a photo that best represented their perspective on the ideal location for placement. The research team used anonymized, de-identified photos and accompanying quotes as a way to share the story and voices of those who would potentially use the machine with the implementation team while maintaining anonymity. This was a modification to the Activate step in the *Our Voice* method that supported the desired anonymity of our community members. The interviewer also asked participants to give feedback on other components of the program, such as additional inventory, registration requirements, and preferences for inventory access (e.g., card, coins, codes).

Using rapid qualitative analysis, the research team summarized our findings following each data collection activity, including the top 5 and bottom 5 locations and key reasons, and a summary of other program feature recommendations [36]. The research team shared results with the implementation team within a few days or weeks after conducting the data collection. Feasibility challenges limited the implementation team’s ability to act on some recommendations made by the participating community members (e.g., locations where an organization was unwilling to partner). In response, the research team took additional steps to further engage with the community to identify opportunities for program alignment with community needs. The research team used methods for community engagement (i.e., surveys, focus groups, and interviews) based on the identified feasibility challenge and logistically feasible opportunities in each community.

## Data collection

Community member responses were audio recorded and professionally transcribed. The research team used meeting notes and field notes to reflect the partnering organizations’ decision-making process. Rapid qualitative analysis was used to capture iterative feedback using a matrix and descriptive summary notes [36]. The research team shared summaries of the results with the implementation team following each data collection period or when potential feasibility challenges were identified, reflecting the iterative nature of user centered design methodology. The research team supplemented summaries with pictures and quotes as a way to share the voice of participants with the implementation team.

## EASY-OPS case examples at 3 participating sites

### Urban integrated safety-net institution

We conducted EASY OPS in our safety net health system from January 2023 to January 2025. Walking audits took place from October 2023 to December 2023. The health system is located on a large campus covering 6 blocks, 0.079 square miles in an urban area.

*Location evaluation*: The implementation team identified the entire urban campus as a potential placement space during feasibility alignment with the research team. During walking interviews with the research team interviewer, initial participant data collection (*n* = 3) consistently identified outdoor locations—such as parking garages and the area near the addiction services building—as preferred, while the emergency department was the least favored due to visible security guards. Security cameras were commonly observed on campus, but participants did not view these as a deterrent for use. Participants identified high foot traffic as a deterrent, highlighting privacy as a key concern. One participant initially preferred a site at the campus edge, but later deprioritized it due to poor sidewalk access and ongoing construction. Instead, they chose the addiction services building, stating, “there are people like me here.” The research team’s summary of results to the implementation team included this quote and image, highlighting the value of walking interviews to capture in-person perceptions.

Subsequent participant data collection (n = 4) with the research team interviewer confirmed similar preferences and introduced an outpatient clinic as a feasible indoor option. Participants reported this location offered privacy, later hours, and accessibility. Similarly, participants noticed security cameras across campus but did not perceive them as a deterrent to use. Participants also suggested that the machine should offer hygiene kits (e.g., soap, menstrual products).

**Fig. 2:**
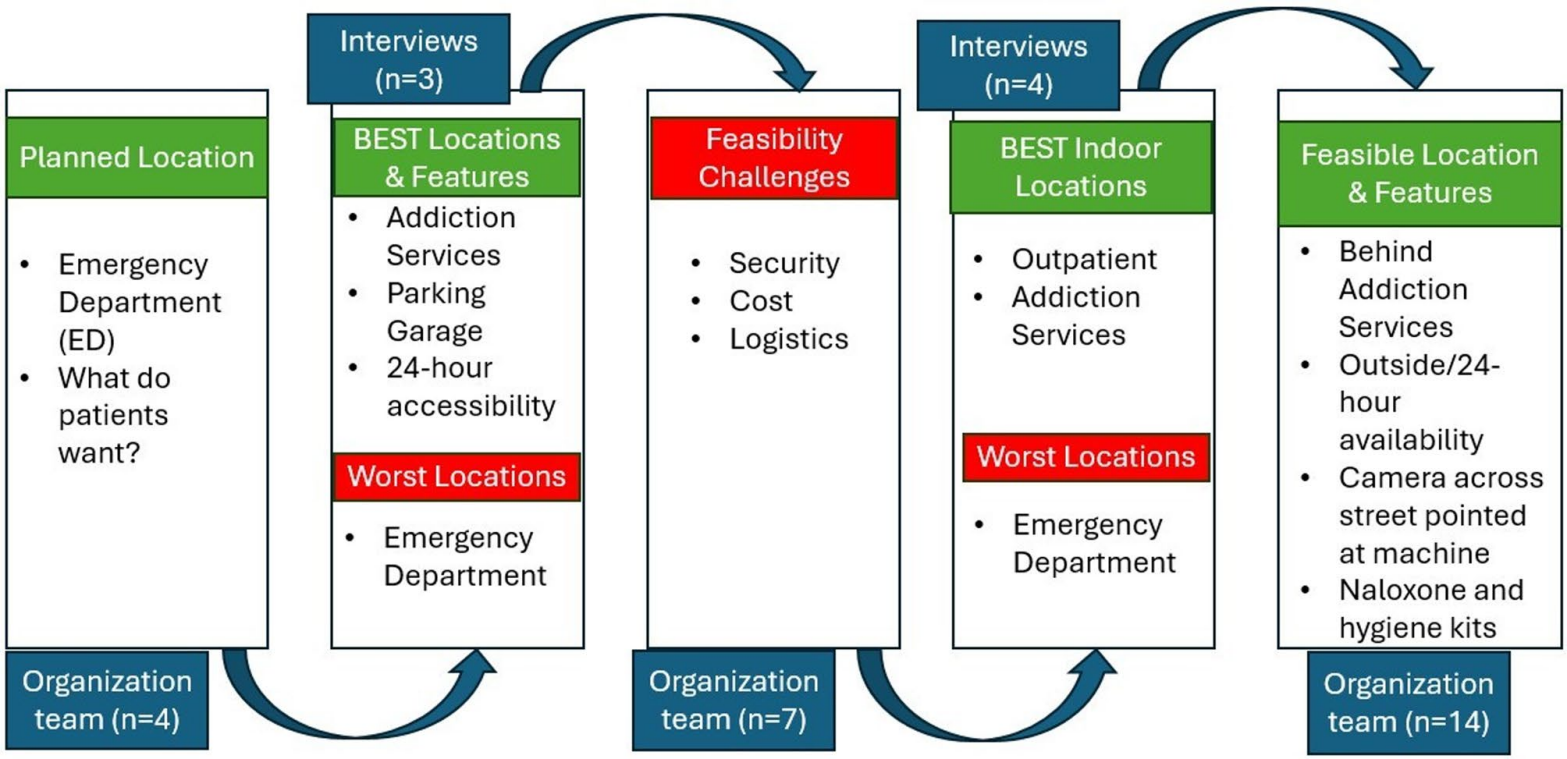
Iterative program development in integrated safety-net health institution

*Iterative Community Engagement* (see Fig. 2): Initially, the implementation team proposed the emergency department for its 24-hour access and high traffic. However, early interview findings with community members revealed a preference for outdoor placement, prompting a reevaluation. Organization leadership (from pharmacy, substance use, security, and engineering departments) identified practical barriers—such as the costs of temperature-controlled units and the need for electrical and concrete infrastructure—along with security concerns at the machine, shifting the implementation team’s focus to indoor options. The research team modified the walking audit guide to address this feasibility concern and the next 4 participants interviewed were also asked to consider ideal indoor locations (“if the machine had to be placed indoors, where is the best location?”). These 4 participants provided consistent suggestions: the outpatient clinic and the addiction services building, both of which offered social comfort and perceived privacy.

The implementation team pulled all department leaders from across the organization together to finalize a location decision. The leadership and research team walked site options and selected an area behind the addiction services building that offered privacy, bus access, security camera coverage, and nearby electrical connections. Despite outdoor placement challenges, the implementation team adapted their plans to reflect community feedback. The leadership and implementation team’s final decisions incorporated input from participants of diverse backgrounds (see Table 1), ensuring the program met the needs of those it aimed to serve.

**Table 1:** Demographics and drug use history of community member participants

Total N	Urban safety net	Rural mountain town	Urban harm reduction agency		All sites
	7	6	8	5	26
Engagement method type	Interview	Interview (n=1) Surveys (n=5)	Interview	Focus group	All
*Age category*					
18–25	0	2	0	0	2
26–45	5	3	6	3	17
46–64	1	1	1	1	4
Unknown	1	0	1	1	3
*Racial categories*					
American Indian/Alaska Native	1	0	3	1	5
Black or African American	2	0	1	0	3
White	4	5	2	2	13
More than one race	0	1	2	1	4
Unknown or not reported	0	0	0	1	1
Ethnic categories					
Hispanic or Latino	0	0	6	4	10
Not Hispanic or Latino	7	6	2	1	16
*Gender*					
Male	5	1	4	1	11
Female	2	5	4	4	15
Unknown	0	0	0	0	0
*Overdose experience*					
Witnessed an overdose	7	2	8	5	22
Experienced an overdose	2	0	2	4	8
Ever obtained naloxone	5	4	7	5	21
Previously used naloxone	4	1	6	5	16
*Current housing*					
My own home or apartment	2	6	0	0	8
Someone else's home or apartment	0	0	5	1	6
Other (includes shelter, halfway house, rehab)	3	0	1	0	4
On the streets	0	0	2	3	5
Prefer not to answer/missing	2	0	0	1	3
*Household monthly income*					
$417 or less	1	0	4	1	6
$418–$1251	0	0	3	2	5
$1251–$2500	3	3	0	0	6
$2501–$3333	1	1	1	0	3
$3334–$6250	1	0	0	1	2
$6251 or more	0	2	0	0	2
Prefer not to answer	1	0	0	1	2
*Any drug use in the last year*…					
Alcohol	3	6	3	4	16
Tobacco	5	3	8	5	21
Prescription drugs for non-medical reasons	6	3	5	3	17
Illegal drugs	7	6	8	5	26
*Any drug use In the last 3 months*…					
Cannabis	5	4	5	4	18
Cocaine	2	5	1	1	9
Prescription stimulants	4	2	2	2	10
Methamphetamine	4	1	7	5	17
Sedatives or sleeping pills	2	3	3	1	9
Hallucinogens	1	6	2	2	11
Street opioids	5	1	5	4	15
Prescription opioids	5	0	5	5	15

### Rural mountain town (tourist destination)

The research team conducted EASY OPS in partnership with a public health department in a tourist mountain town from May 2023 to June 2024, with machine placement continuing through November 2024. The region included 9 towns across 608 square miles. The interviewer conducted a walking audit in October 2023. The research team used online surveys as a way to address recruitment barriers from February 2024 to May 2024.

*Location evaluation*: The implementation team identified the full county as an option for placement during feasibility alignment with the research team. The first participant interviewed by the research team identified transit stops, employee housing near ski resorts, seasonal worker apartments, and entertainment venues (bars, restaurants, music venues) as ideal sites. The participant identified locations connected to public service, including law enforcement and fire departments where naloxone is currently distributed, that would not be used. The participant noted “for me, associating with the government doesn’t feel safe.” The participant also flagged connectivity issues (Wi-Fi/cell signal) as barriers to electronic registration and outdoor placement. Anonymous survey participants from the rural community identified housing, transit stops, and entertainment venues as top locations for placement similar to the initial interviewee. Survey participants also suggested placement at health service locations.

**Fig. 3:**
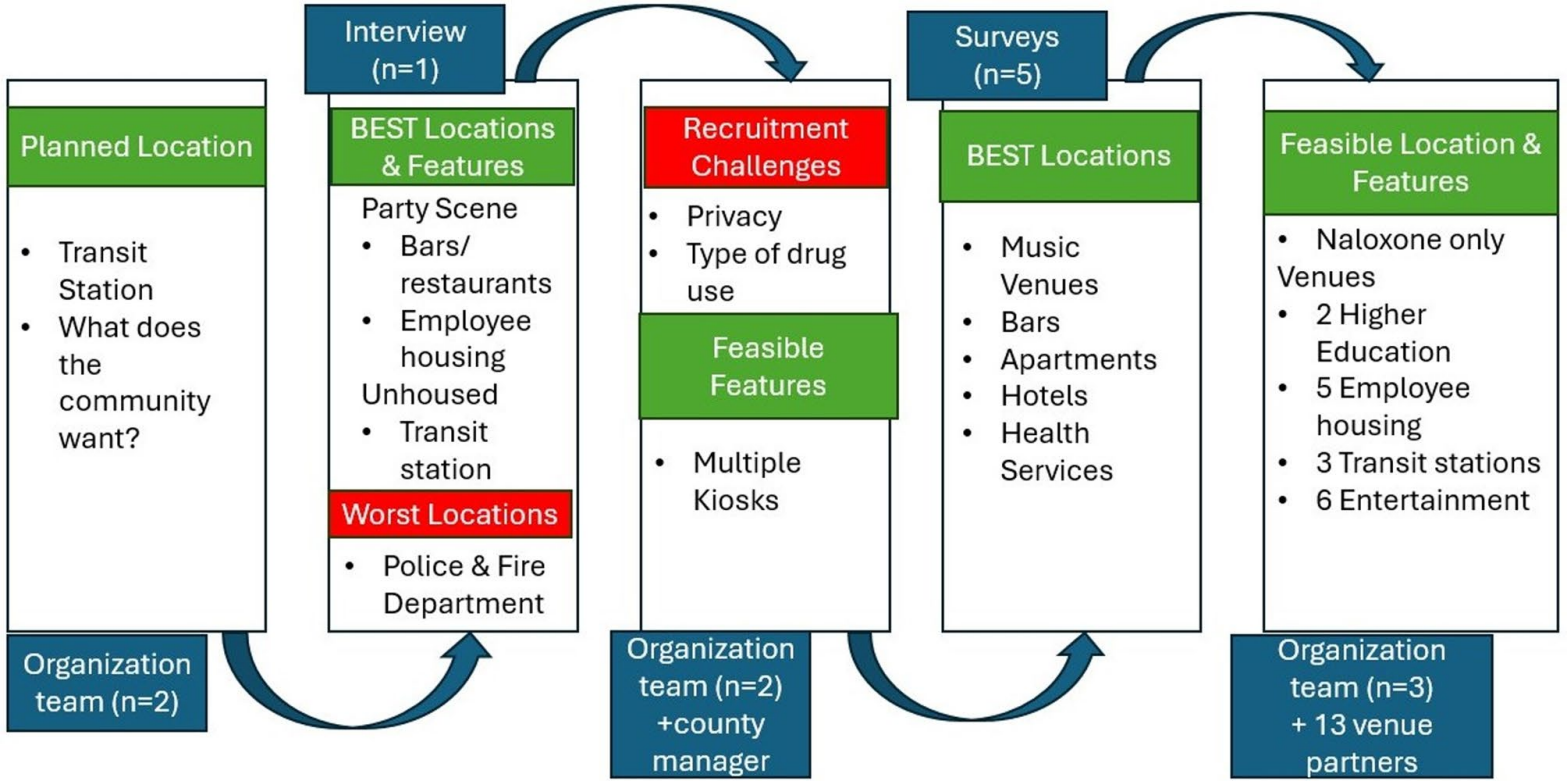
Iterative program development in rural mountain public health department

*Iterative Community Engagement* (see Fig. 3): The implementation team initially prioritized transit stations. After low recruitment at clinics, the research team spent two weeks in town to recruit during methadone and community clinic hours. One participant, already in a community advisory role, noted two distinct populations: unhoused individuals and a party-scene demographic. Locals shared potential recruitment barriers including concerns that the research team might be affiliated with law enforcement and observations that stimulant use was more common than opioids. In response, the research team broadened eligibility criteria to substances that may contain opioids and emphasized confidentiality, though we still were unable to obtain additional participants.

The implementation team pivoted from vending machines to naloxone kiosks (newspaper box style), which were lower cost and allowed broader distribution to support the differing population needs. Securing placement partners was time-intensive for the implementation team, but the initial interview helped narrow down the list to improve efficiency. To expand input and help further narrow the list of potential location partners, the research team shifted interview questions into a survey format that could be taken in English or Spanish, anonymously, or with a $25 gift card if the participant chose to share an email address. The implementation and research team placed flyers throughout the community and engaged with organizations serving people who use substances. Potential participants opened 37 surveys, 13 of whom were ineligible to complete the survey (age, no illegal drug use, and not residing in the county), 17 were started with missing data, and 7 participants completed surveys. The research team removed two of the completed 7 surveys due to poor data quality, leaving a total of 5 complete responses. Key location types for desired placement remained consistent: housing, transit, and entertainment venues. Compared to interviews and focus groups, the survey reached a younger, more stimulant-using, and stably housed population with higher incomes (see Table 1).

Guided by community input, the implementation team focused on transit stations, employee housing, entertainment venues, and partnership with a local community college. Ultimately, the implementation team placed 16 naloxone kiosks in transit stations, higher education sites, entertainment venues (bars, restaurants, music venues, coffee shop), and employee housing—decisions directly shaped by community engagement and feedback.

### Urban harm reduction agency

The research team conducted EASY OPS in our urban harm reduction agency from July 2023 to January 2025 (note: second site went live March 2025) serving predominantly Hispanic/Latinx populations. The research team spent one week in February 2024 dedicated to conducting walking audits (N = 8) and 1 focus group (N = 5). The agency operates in a 56 square mile urban center.

*Location evaluation*: The implementation team identified the entire city as a placement option during feasibility alignment. Over 3 days the research team interviewer completed 8 data collection activities (Discovery Tool walks) with community members. Themes included a neighborhood in which there are no harm reduction services, a central location people often walk through on their way to other neighborhoods, near the harm reduction agency, near homeless encampments, and accessibility in the evenings. Participants identified parks and gas stations as key locations for placement due to reported frequent drug use and areas where PWLE congregate. As the interviewers drove the community with participants, participants consistently identified one gas station in a neighborhood lacking other support services. One participant noted “you got-you got people that sittin’ on there, sittin’ on the side of the (convenience store), sittin’ behind it gettin’ high….I mean, and those people are there all night long.”

**Fig. 4:**
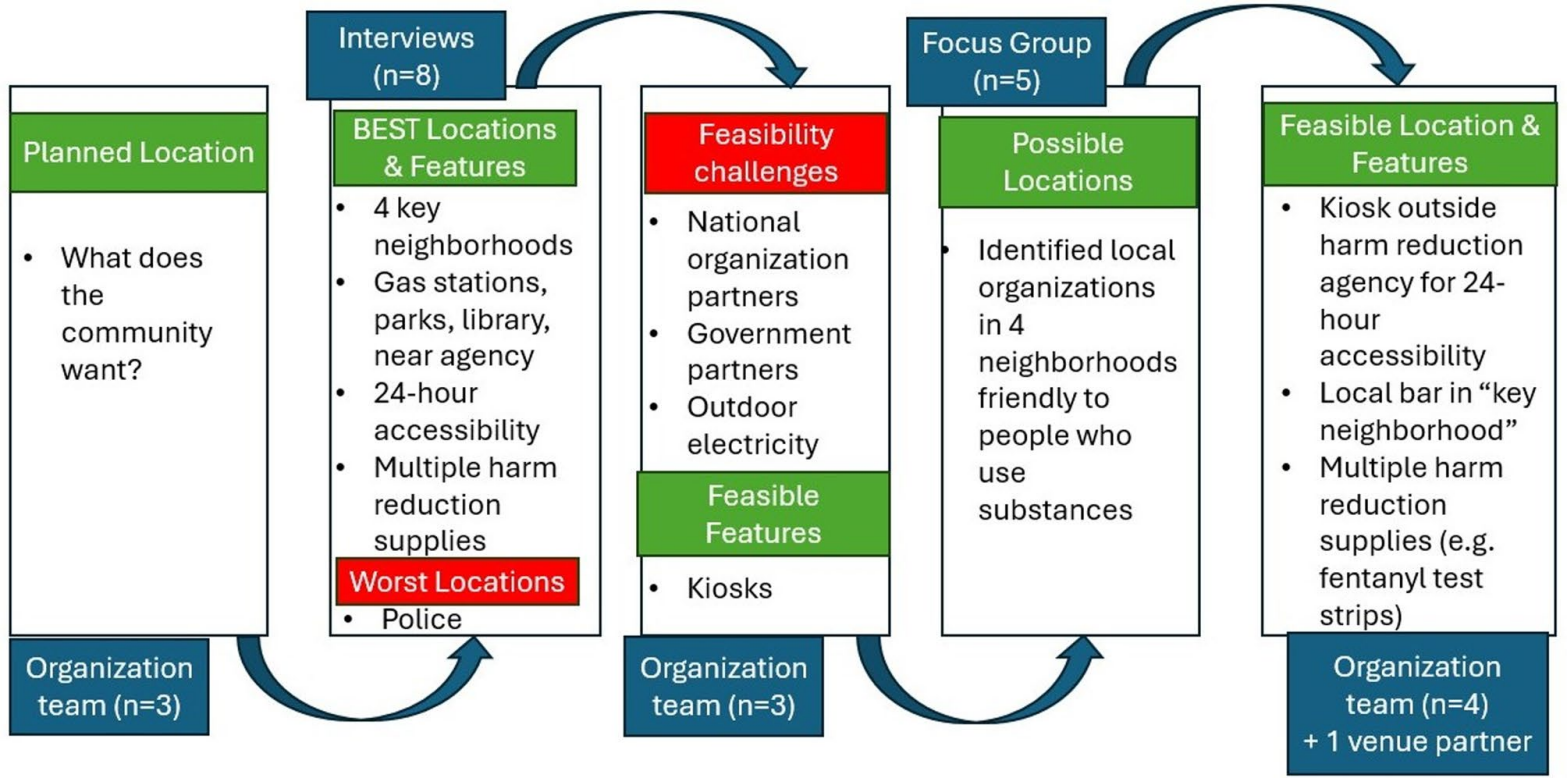
Iterative program development in urban harm reduction agency

*Iterative community engagement* (see Fig. 4): The research team presented results of the initial interviews to the implementation team who revealed feasibility challenges due to the local political climate, hindering the placement of machines in parks and transit stations. Additionally, partnering with national organizations like gas stations posed a feasibility challenge.

To address these feasibility concerns, the research team went back to community members for additional feedback. We used time during the weekly syringe service program to recruit participants for a focus group discussion (N = 5). In the focus group, the interviewer asked participants to consider local organizations in the identified neighborhoods that may be friendly to harm reduction services for machine placement partnership. The participants identified several businesses with which to explore machine placement. The implementation team used this information to reach out to potential business partners, starting in the neighborhoods currently lacking harm reduction services.

The implementation team was able to place a machine in a local bar along one of the walking paths in the central neighborhood identified by participants, and another machine outside the harm reduction agency for 24-hour access to supplies. The implementation team continues ongoing conversations with the library and other local organizations in the neighborhood lacking services, with plans for expansion following the initial pilot test.

## Conclusion

Our findings from the EASY OPS approach highlight the importance of directly involving PWLE in implementing harm reduction programs, in order to identify environmental factors that affect access for intended end-users. Without community input, the research and implementation teams would have missed key locations in each of the communities involved. In our health system, community members identified the original location planned for placement as the worst location due to a social environment involving security guards at the door which was perceived as threatening. In our harm reduction agency, participants helped the implementation team identify a critical neighborhood lacking services and a need for accessibility near the harm reduction agency outside of regular business hours. In addition, locations identified by focus group members supported the harm reduction agency in building partnerships with their community. Active “on the ground” data collection and surveys in our rural community highlighted the need to focus on the entertainment industry, which was not a key target for machine placement originally.

Another key finding from the EASY OPS approach is that it operationalizes a process for ongoing collaboration between program implementers and potential end-users facilitated by the research team. This helps balance feasibility constraints with end-users’ priorities for environmental and program features to improve harm reduction services. Related to the need for efficient engagement with PWLE, it offers opportunities to give feedback at different phases of program development and was flexible for our participating PWLE. This type of approach, which does not require longitudinal and consistent involvement, is promising for PWLE who may be less willing or able to participate in ongoing research collaborations. The flexibility allowed for more efficiency, requiring as little as one week in total in our harm reduction location. We also found in our work that modifying strategies to provide opportunities for anonymous input also increased our research team’s ability to obtain information in communities where substance use services and trust in public systems were lacking. Community members’ iterative feedback supported organizational decision making while balancing feasibility challenges for sustainable programs that met the needs of the community.

Our findings highlight the value of obtaining broad feedback from community members and not limiting them to only perceived feasible options. In the case of our health system site, our implementation team did not believe outdoor locations were feasible at first. However, due to the feedback we obtained in interviews, the implementation and leadership teams were able to find solutions to the feasibility challenges of an outdoor location and meet the needs of our community members. Similarly, we originally planned to use vending machines, but many of the locations identified by our interview participants at both the rural mountain location and urban location served by the harm reduction agency did not have capacity for electricity. By switching from vending machines to kiosks programs, the implementation teams’ decisions better aligned with community members’ desired placement locations.

CBPR work includes a spectrum of engagement with community members [15]. On one side of the spectrum, researchers can do important work with community members in which the community perspective drives research questions and advocates for needs in a specific community. Dr. King and colleagues are positively impacting communities throughout the world through using the *Our Voice* method to give voice to citizen scientists as positive change agents in partnership with researchers, stakeholders, and local decision-makers [37]. However, that level of community participation is not always feasible due to time, funding, and participation constraints [17, 18, 21]. When organizations are implementing evidence-based practices, or community members are unwilling or unable to participate in ongoing research collaboration, EASY OPS and other innovative strategies to include community input can be used to support harm reduction program development that is responsive to PWLE. The EASY OPS iterative approach to engagement creates flexibility to incorporate feedback as needed to address contextual challenges. As we found in our rural mountain town, people were unwilling to participate in interviews, but responded to a survey when given the option for anonymity and easier access feedback channels. In our urban harm reduction agency, a focus group helped the implementation team identify new potential locations to partner with that would reach the community members who needed it. In our urban health center, summaries, pictures, and quotes of participant feedback shifted the implementation and leadership team’s perspective, who had had concerns about logistics and security. The implementation and leadership team identified ways in which they could accommodate 24-hour accessibility with security cameras and engineering options to ensure those who need the program would use it.

While this project displayed diversity in locations and settings, it was part of a pilot test and not designed to formally evaluate the effectiveness of the implementation process. The research team did not assess community engagement and support of the final program. Next steps for this program include formal evaluation and assessment of community perspectives.

It is well-established that involving individuals in the research process for whom interventions are designed is crucial to ensuring programs reflect the priorities of the communities they aim to serve [38, 39]. While ongoing community-engaged research may be ideal and builds a community in support of the project, it may not always be feasible. Alternate strategies to achieve ongoing community input to inform the development process can pose a critical addition to community-engaged research by obtaining underrepresented perspectives when endeavoring to maximize equitable outcomes.

## Data Availability

The datasets used and/or analyzed during the current study are available from the corresponding author on reasonable request.

## Abbreviations

OEND: Overdose education and naloxone distribution
EBP: Evidence based practice
PWLE: People with lived and living experience
HRVM: Harm reduction vending machine
VENDY: VEnding machine Naloxone Distribution in Your community
UCD: User centered design
EASY OPS: Effective adaptable and sustainable in your community: operationalizing program sustainability

## References

[1] Des Jarlais DC. Harm reduction in the USA: the research perspective and an archive to David Purchase. Harm Reduct J. 2017;14(1):1–7. 28747189 10.1186/s12954-017-0178-6 PMC5530540

[2] Platt L, Minozzi S, Reed J, Vickerman P, Hagan H, French C, et al. Needle syringe programmes and opioid substitution therapy for preventing hepatitis C transmission in people who inject drugs. Cochrane Database Syst Rev. 2017;9:CD012021. 10.1002/14651858.CD012021.pub2. 28922449 10.1002/14651858.CD012021.pub2 PMC5621373

[3] McDonald R, Strang J. Are take-home naloxone programmes effective? Systematic review utilizing application of the Bradford Hill criteria. Addiction. 2016;111(7):1177–87. 27028542 10.1111/add.13326 PMC5071734

[4] Fernandes RM, Cary M, Duarte G, Jesus G, Alarcão J, Torre C, et al. Effectiveness of needle and syringe Programmes in people who inject drugs–An overview of systematic reviews. BMC Public Health. 2017;17(1):1–15. 28399843 10.1186/s12889-017-4210-2 PMC5387338

[5] Wood E, Tyndall MW, Qui Z, Zhang R, Montaner JS, Kerr T. Service uptake and characteristics of injection drug users utilizing North America’s first medically supervised safer injecting facility. Am J Public Health. 2006;96(5):770–3. 16571703 10.2105/AJPH.2004.057828 PMC1470579

[6] Wilson DP, Donald B, Shattock AJ, Wilson D, Fraser-Hurt N. The cost-effectiveness of harm reduction. Int J Drug Policy. 2015;26:S5–S11. 25727260 10.1016/j.drugpo.2014.11.007

[7] Bruzelius E, Cerdá M, Davis CS, Jent V, Wheeler-Martin K, Mauro CM, et al. Naloxone expansion is not associated with increases in adolescent heroin use and injection drug use: evidence from 44 US states. Int J Drug Policy. 2023;114:103980. 36863285 10.1016/j.drugpo.2023.103980 PMC11268161

[8] Binswanger I, et al. Naloxone co-dispensing with opioids: a randomized pragmatic trial. J Gen Internal Med. 2022;37:2624–33. 10.1007/s11606-021-07356-6 PMC9411391 35132556

[9] Kral AH, Lambdin BH, Wenger LD, Davidson PJ. Evaluation of an unsanctioned safe consumption site in the United States. N Engl J Med. 2020;383(6):589–90. 32640126 10.1056/NEJMc2015435

[10] Coffin PO, Sullivan SD. Cost-effectiveness of distributing naloxone to heroin users for lay overdose reversal. Ann Intern Med. 2013;158(1):1-U42. 23277895 10.7326/0003-4819-158-1-201301010-00003

[11] Lopez AM, Thomann M, Dhatt Z, Ferrera J, Al-Nassir M, Ambrose M, et al. Understanding racial inequities in the implementation of harm reduction initiatives. Am J Public Health. 2022;112(S2):S173–S181. 35349311 10.2105/AJPH.2022.306767 PMC8965181

[12] Lambdin BH, Zibbell J, Wheeler E, Kral AH. Identifying gaps in the implementation of naloxone programs for laypersons in the United States. Int J Drug Policy. 2018;52:52–5. 29232604 10.1016/j.drugpo.2017.11.017

[13] Bandara S, Byrne L, Berman V, Hurst A, King D, Gibbons JB, et al. Harm reduction and treatment among people at high risk of overdose. JAMA Netw Open. 2024;7(8):e2427241. 10.1001/jamanetworkopen.2024.27241 PMC11320172 39133486

[14] Collins SE, Clifasefi SL, Stanton J, Straits KJ, Gil-Kashiwabara E, Rodriguez Espinosa P, et al. Community-based participatory research (CBPR): towards equitable involvement of community in psychology research. Am Psychol. 2018;73(7):884. 29355352 10.1037/amp0000167 PMC6054913

[15] Ramanadhan S, Davis M, Donaldson ST, Miller E, Minkler M. Participatory approaches in dissemination and implementation science. Dissem Implement Res Health Transl Sci Pract. 2023;212:231.

[16] Kwon SC, Tandon SD, Islam N, Riley L, Trinh-Shevrin C. Applying a community-based participatory research framework to patient and family engagement in the development of patient-centered outcomes research and practice. Transl Behav Med. 2018;8(5):683–91. 30202926 10.1093/tbm/ibx026 PMC6128966

[17] De Las Nueces D, Hacker K, DiGirolamo A, Hicks LS. A systematic review of community-based participatory research to enhance clinical trials in racial and ethnic minority groups. Health Serv Res. 2012;47(3pt2):1363–86. 22353031 10.1111/j.1475-6773.2012.01386.x PMC3418827

[18] Vangeepuram N, Fei K, Goytia C, Madden D, Corbie-Smith G, Horowitz CR. Community-based participatory research: insights, challenges, and successes from the perspectives of frontline recruiters and investigators. J Particip Res Methods. 2023;4(2). 10.35844/001c.77399.

[19] Brown G, Crawford S, Perry G-E, Byrne J, Dunne J, Reeders D, et al. Achieving meaningful participation of people who use drugs and their peer organizations in a strategic research partnership. Harm Reduct J. 2019;16(1):1–10. 31182099 10.1186/s12954-019-0306-6 PMC6558880

[20] Hoffman KA, Baker R, Kunkel LE, Waddell EN, Lum PJ, McCarty D, et al. Barriers and facilitators to recruitment and enrollment of HIV-infected individuals with opioid use disorder in a clinical trial. BMC Health Serv Res. 2019;19(1):862. 31752905 10.1186/s12913-019-4721-x PMC6868733

[21] Souleymanov R, Kuzmanović D, Marshall Z, Scheim AI, Mikiki M, Worthington C, et al. The ethics of community-based research with people who use drugs: results of a scoping review. BMC Med Ethics. 2016;17(1):25. 27129927 10.1186/s12910-016-0108-2 PMC4850694

[22] Mathias H, Duff E, Schulz P, Auger S, Gravel-Ouellette A, Lockhart T, et al. Rural community-based participatory research with families of people who use drugs: key considerations from a multi-provincial research partnership. Harm Reduct J. 2025;22(1):92. 40448147 10.1186/s12954-025-01247-3 PMC12124088

[23] Nilsen P. Making sense of implementation theories, models and frameworks. Implement Sci. 2015;10:53. 25895742 10.1186/s13012-015-0242-0 PMC4406164

[24] Lister JJ, Weaver A, Ellis JD, Himle JA, Ledgerwood DM. A systematic review of rural-specific barriers to medication treatment for opioid use disorder in the United States. Am J Drug Alcohol Abuse. 2020;46(3):273–88. 31809217 10.1080/00952990.2019.1694536

[25] Carlson J, Dean K, Sallis J. Measures registry user guide: physical activity environment. Washington (DC): Natl Collab Child Obes Res. 2017. https://nccororgms.wpengine.com/tools-mruserguides/wp-content/uploads/sites/2/2017/NCCOR_MR_User_Guide_Physical_Activity-FINAL.pdf.

[26] Wagner NM, Kempe A, Barnard JG, Rinehart DJ, Havranek EP, Glasgow RE, et al. Qualitative exploration of public health vending machines in young adults who misuse opioids: a promising strategy to increase naloxone access in a high risk underserved population. Drug Alcohol Dependence Rep. 2022. 10.1016/j.dadr.2022.100094. 10.1016/j.dadr.2022.100094 PMC9851265 36687307

[27] Russell E, Johnson J, Kosinski Z, Kaplan C, Barnes N, Allen S, et al. A scoping review of implementation considerations for harm reduction vending machines. Harm Reduct J. 2023;20(1):33. 36927354 10.1186/s12954-023-00765-2 PMC10018614

[28] Zhang A, Carrillo M, Liu R, Ballard SM, Reedy-Cooper A, Zgierska AE. Vending machines for reducing harm associated with substance use and use disorders, and co-occurring conditions: a systematic review. Harm Reduct J. 2025;22:89. 40437578 10.1186/s12954-025-01236-6 PMC12121115

[29] Allen ST, O’Rourke A, Johnson JA, Cheatom C, Zhang Y, Delise B, et al. Evaluating the impact of naloxone dispensation at public health vending machines in Clark County, Nevada. Ann Med. 2022;54(1):2692–700. 36168975 10.1080/07853890.2022.2121418 PMC9542801

[30] Maguire M. Methods to support human-centred design. Int J Hum Comput Stud. 2001;55(4):587–634.

[31] Lyon AR, Koerner K. User-centered design for psychosocial intervention development and implementation. Clin Psychol Sci Pract. 2016;23(2):180–200. 10.1111/cpsp.12154 PMC5812700 29456295

[32] Mohr DC, Lyon AR, Lattie EG, Reddy M, Schueller SM. Accelerating digital mental health research from early design and creation to successful implementation and sustainment. J Med Internet Res. 2017;19(5):e7725. 10.2196/jmir.7725 PMC5443926 28490417

[33] Kinzie MB, Cohn WF, Julian MF, Knaus WA. A user-centered model for web site design: needs assessment, user interface design, and rapid prototyping. J Am Med Inform Assoc. 2002;9(4):320–30. 12087113 10.1197/jamia.M0822 PMC346619

[34] King AC, Odunitan-Wayas FA, Chaudhury M, Rubio MA, Baiocchi M, Kolbe-Alexander T, et al. Community-based approaches to reducing health inequities and fostering environmental justice through global youth-engaged citizen science. Int J Environ Res Public Health. 2021;18(3):892. 33494135 10.3390/ijerph18030892 PMC7908382

[35] Buman MP, Winter SJ, Sheats JL, Hekler EB, Otten JJ, Grieco LA, et al. The stanford healthy neighborhood discovery tool: a computerized tool to assess active living environments. Am J Prev Med. 2013;44(4):e41–7. 23498112 10.1016/j.amepre.2012.11.028 PMC3601583

[36] Hamilton AB, Finley EP. Qualitative methods in implementation research: an introduction. Psychiatr Res. 2019;280:112516. 10.1016/j.psychres.2019.112516 PMC7023962 31437661

[37] Pedersen M, Wood GE, Fernes PK, Goldman Rosas L, Banchoff A, King AC. The “our voice” method: participatory action citizen science research to advance behavioral health and health equity outcomes. Int J Environ Res Public Health. 2022;19(22):14773. 36429494 10.3390/ijerph192214773 PMC9690580

[38] Huebschmann AG, Brega AG, Stotz SA, Shane AL, King R, Jernigan VBB, et al. Gaps and opportunities for measuring equity with the Translational Science Benefits Model: recommendations from the Center for American Indian and Alaska Native Diabetes Translation Research. J Clin Transl Sci. 2024;8(1):e206. 39655025 10.1017/cts.2024.638 PMC11626608

[39] Fort MP, Manson SM, Glasgow RE. Applying an equity lens to assess context and implementation in public health and health services research and practice using the PRISM framework. Front Health Serv. 2023;3:1139788. 37125222 10.3389/frhs.2023.1139788 PMC10137153

